# Challenges and opportunities related to penicillin allergy in the Veterans Health Administration: a narrative review

**DOI:** 10.1017/ash.2023.448

**Published:** 2023-10-19

**Authors:** Marcus A. Kouma, Jessica M. Guastadisegni, Linda Yang, Daniel N. Maxwell, Donald F. Storey, Reuben J. Arasaratnam

**Affiliations:** 1 Veterans Affairs North Texas Health Care System, Dallas, TX, USA; 2 Veterans Affairs North Texas Health Care System and University of Texas Southwestern Medical Center, Dallas, TX, USA

## Abstract

The presence of a penicillin allergy label in a patient’s medical chart is associated with negative clinical and economic outcomes. Given that less than 10% of reported reactions are truly immunoglobulin E-mediated, removal of unverified penicillin allergy labels is a public health priority and an area of ongoing implementation research. The Veterans Health Administration (VHA) is the largest integrated healthcare system in the United States, with almost 9 million veterans currently enrolled. However, studies analyzing the impact of the penicillin allergy label in this population are limited to single facilities and largely focus on short-term outcomes of allergy documentation correction, usage of β-lactams, and avoidance of antibiotic-related side effects. Broader, national VHA studies focusing on health outcomes and costs are lacking. As with non-VHA facilities, penicillin allergy evaluations are limited owing to the absence of formal allergy/immunology services at most VHA facilities. Pharmacy-driven screening and referral for clinic-based penicillin skin testing is a promising and frequently discussed modality in the literature, but its scalability within the VHA is not yet proven. Broader, evidence-based strategies that can be adapted to the available resources of individual VHA facilities, including those without on-site access to allergy providers, are needed.

## Introduction

With a reported prevalence of 6%–25%, penicillin allergy is the most frequently reported drug allergy worldwide.^1^ However, data suggest that less than 10% of patients with a penicillin allergy label in their medical charts have a true immunoglobulin E-mediated hypersensitivity.^2,3^ Erroneous documentation of penicillin allergy is a public health threat and is associated with receipt of less appropriate alternative antibiotic therapy, increased health care costs, longer hospital stays, development of drug-resistant infections, and increased mortality.^4,5^ In the United States, the American Academy of Allergy, Asthma, and Immunology (AAAAI); the American College of Allergy, Asthma, and Immunology (ACAAI); the Centers for Disease Control and Prevention, Society for Healthcare Epidemiology of America; and the Infectious Diseases Society of America have initiated efforts to promote recognition and removal of inaccurate penicillin allergy labels. Several resources are available to assist in the evaluation and removal of penicillin allergy.^6,7^ In a recent guidance document “Drug allergy: A 2022 practice parameter update,”^8^ the AAAAI and ACAAI support proactive penicillin allergy delabeling and the use of direct amoxicillin challenge in patients with low-risk penicillin allergy histories (defined as benign cutaneous reactions occurring more than 5 yr ago). Allergy societies outside the United States have endorsed similar approaches, including the important role of non-allergy specialists in delabeling services.^9,10^

The Veterans Health Administration (VHA) is the largest integrated healthcare system in the United States, with approximately 9 million veterans currently enrolled in care.^11^ Advancing age and high prevalence of chronic comorbidities increase veterans’ risks of bacterial infections and subsequent need for antibiotic therapy.^12^ Additionally, the VHA performs over 600,000 surgical procedures annually^13^ for which β-lactams are the preferred prophylactic agents.^14^ This magnifies the importance of identifying and removing unverified penicillin allergy labels specifically in the US veteran population.

As infectious diseases practitioners actively involved in penicillin allergy evaluation, the authors conducted a narrative review (methods detailed in Supplementary Appendix 1 and results shown in Tables 1 and 2) on the current state of penicillin allergy within the VHA and addressed ongoing challenges and opportunities for growth.

**Table 1.** Epidemiology of penicillin allergy within VHA

Study	Setting and population	Methodology	Major findings	Additional comments
Neu et al., 2022^17^	Setting: Memphis VAMC Population: Veterans (*n* = 95) with documented β-lactam allergy who were admitted and received antibiotics.	Retrospective, observational analysis over 6 yr (2011–2016).	In total, 48% of patients received nonpreferred antibiotics, 67% of which were inappropriate. Number of ADEs in the nonpreferred group was statistically greater than the number of ADEs in the preferred group (12 vs 2, *P* < .01). There were more patients with at least 1 negative outcome on nonpreferred therapy than those on preferred therapy (23 vs 11, *P* < .01).	
Vivo et al., 2022^37^	Setting: VA dental clinics Population: Adults (*n* = 300) with documented non-anaphylactic penicillin allergy who did not have an oral infection within 1 wk of their visit and who received an antibiotic from a dentist.	Retrospective cross-sectional analysis over 3 yr (2015–2018).	In total, 53% of patients qualified for penicillin skin testing and 27% for skin testing or oral penicillin challenge. 10% of patients had a pseudoallergy and 20% did not have any documented reaction.	In total, 5.6% of patients with history of moderate-to-high-risk or high-risk reactions to penicillin received a penicillin-class antibiotic.
Meledathu et al., 2021^a^	Setting: Washington DC VAMC Population: Veterans (*n* = 305) that underwent a total knee or hip arthroplasty	Retrospective chart review over 3 yr (2018–2020)	All 26 patients with a penicillin allergy received a non-β-lactam agent: 62% received vancomycin and 29% received clindamycin. Only one patient had an allergy consult, but the penicillin allergy was not addressed at the visit.	Facility initiated a penicillin delabeling program in 2018 including skin testing and oral challenge in collaboration with Allergy and Immunology.
Strymish et al., 2020^21^	Setting: 109 VA facilities Population: Veterans in the External Peer Review Program who underwent major cardiac, orthopedic total joint replacement, vascular, or colorectal procedures. Included: Database of almost 80,000 procedures.	Retrospective cohort study over 5 yr (2008–2013).	Allergy and/or intolerance to β-lactam was the second most common reason for vancomycin administration as preoperative prophylaxis (34%), with perception of high facility MRSA rate being the most common (46.9%). Risk for acute kidney injury was lower in patients who received β-lactam compared with patients who received vancomycin as preoperative prophylaxis (RR = 0.7 for ortho and 0.88 for cardiac, *P* < .001).	A β-lactam allergy was the most common reason for vancomycin use for colorectal and vascular procedures.
Mason et al., 2019^19^	Setting: VA Western New York Healthcare System Population: Adults discharged on selected oral antibiotics with adequate chart documentation to assess the appropriateness of therapy. Included: 1,884 antibiotic prescriptions (221 with β-lactam allergy, 1,623 without β-lactam allergy).	Retrospective chart review during an 8-mo period (June 2017–February 2018).	In total, 34.8% of patients with β-lactam allergy received a fluroquinolone compared with 19.5% of patients without the allergy. Patients with β-lactam allergy were 31% less likely to be prescribed the appropriate antibiotic based on indication.	In total, 10.9% of patients with β-lactam allergy received a β-lactam with no adverse reactions.
Ness et al., 2019^18^	Setting: Memphis VAMC Population: Veterans (*n* = 80) with β-lactam allergy prescribed an antibiotic from an outpatient clinic or ED.	Retrospective chart review over 6 yr (2011–2016).	In total, 57% of antibiotic courses prescribed were nonpreferred, and of these, 56% were inappropriate. In total, 39% of patients safely received a β-lactam antibiotic after documentation of an allergy.	There was no correlation between negative outcomes and receipt of nonpreferred or inappropriate antibiotics.
Conway et al., 2017^16^	Setting: VA Western New York Healthcare System Population: Veterans (*n* = 403) admitted through the ED with a diagnosis of pneumonia, urinary tract infection, bacteremia, or sepsis.	Retrospective chart review over 10 yr (2006–2015).	In total, 14.1% of patients had a penicillin allergy, with the most frequently documented reaction as unknown at 33.3% and only 8.8% as severe. Patients with a penicillin allergy were more likely to receive a fluoroquinolone (61.4% vs 26.3%, *P* < .0001) or carbapenem (5.3% vs 0.3%, *P* < .0001) than patients without a penicillin allergy. There was a 50-min delay to first dose of antibiotics for patients with a penicillin allergy compared with patients without a penicillin allergy.	Patients with penicillin allergy were 18% less likely to have their antibiotic regimen de-escalated (*P* = .01). Patients in both groups had similar total duration of antibiotics, rate of IV to PO conversion, length of stay, and 30-d readmission rate.
McConeghy et al., 2017^15^	Setting: VAMCs Population: Any inpatient admission and the linked allergy history. Included: 10.8 million inpatient admissions.	Retrospective cohort study over 15 yr (2000–2014).	The most common allergy was penicillin at 13%, with most documented reaction being rash at 25.5%. 43.3% of penicillin allergies did not have a documented reaction.	Compared to other drug classes, the percentage of admissions with penicillin or sulfonamide allergy remained stable over the study period, with an increase of 1% or less.
Biagtan et al., 2014^20^,^a^	Setting: Middleton VAMC Population: Veterans with a penicillin allergy reported in 2008. Included: 102 patients with 352 outpatient antibiotic orders.	Retrospective chart review over 4 yr (2009–2012).	Penicillin-allergic patients had a 60% greater average estimated antibiotic cost compared with patients without a penicillin allergy.	
Ponce et al., 2014^41^	Setting: 94 VA facilities Population: Veterans undergoing elective hip or knee arthroplasty. Included: 18,830 procedures.	Retrospective cohort study over 5 yr (2005–2009)	The presence of a penicillin allergy was not associated with differences in the rates of surgical site infections, though it was associated with antibiotic selection. Only smoking and vancomycin monotherapy were found to be independent predictors of SSIs.	Lack of association between PCN allergy and SSI may be affected by low event rates
Wang and Terry, 1971^42^	Setting: Wood VA Center in Milwaukee, WI Population: Veterans (*n* = 8,291) admitted to the hospital while the surveillance program was active.	Surveillance program with a trained nurse observer who reviewed patients in the hospital for any suspected adverse drug reaction over 1 yr (March 1967–February 1968).	In total, 1.54% of patients had an adverse drug reaction. In total, 58.5% of drug reactions were due to antibiotics, with penicillin being the most common group.	

Note. ADE, adverse drug event; ED, emergency department; MRSA, methicillin-resistant *Staphylococcus aureus*; RR, relative risk; VA, Veterans Affairs; VAMC, VA Medical Center.

a Abstract only available.

**Table 2.** Penicillin allergy evaluation within VHA

Study	Setting and population	Methodology and intervention	Major findings	Comments
Gaberino et al., 2022^30^	Setting: VAMC in Milwaukee, WI. Population: Adults (inpatients and outpatients) with a history of penicillin or cephalosporin allergy.	β-Lactam allergy assessment guideline quality improvement project over 3 yr (2017–2020).	Allergy updated or removed in 48% or 27% of patients, respectively. Significant increases in the percentage of β-lactam antibiotics prescribed for groups with allergy removed or updated.	Use of antibiotic test-dose order sets. Allergy/Immunology participation on multidisciplinary team.
Nguyen et al., 2022^38^	Setting: VA hospital in Los Angeles, CA. Population: Emergency department patients with listed penicillin allergy.	Emergency department Clinic Pharmacy Specialists evaluated patients with penicillin allergy, performed direct amoxicillin challenge on low-risk patients being admitted, and referred ineligible patients to Allergy/Immunology over a 9-mo period.	Of 128 patients who presented to the emergency department, 40 (31%) were directly delabeled based on allergy history, and 16 (13%) were delabeled following oral amoxicillin challenge.	57 patients were referred to Allergy/Immunology, of which 11 were delabeled.
Kakumanu, 2022^43^,^a^	Setting: VA hospital in Madison, WI. Population: Inpatients with listed penicillin allergy.	Pharmacy and antimicrobial stewardship team performed chart reviews over 3 yr, with 382 patients identified.	In total, 11% of patients were directly delabeled during their hospital stay.	Most commonly cited barriers to lack of allergy review were lack of time and lack of electronic medical record tools to review past antibiotic history.
Mitchell et al., 2021^29^	Setting: VAMC in Memphis, TN. Population: Adults admitted with documentation of penicillin allergy in the electronic health record.	β-Lactam allergy assessment quality improvement initiative over 3 yr (2017–2020).	In total, 31% of patients were cleared for penicillin allergy removal. 68% of patients were cleared for alternative β-lactams.	Eligible patients were referred to an ASP/ID pharmacist-run clinic for skin testing and oral amoxicillin challenge. Allergy/immunology supervision of clinic.
Piazza et al., 2021^39,^ ^a^	Setting: VAMC in Miami, FL. Population: Inpatients with penicillin allergy.	Daily report was reviewed and appropriate inpatients were challenged with oral amoxicillin and observed for 60 min. The allergy label was removed for those who had no reaction. Those that were not candidates for oral challenge were offered outpatient PST.	In total, 39 patients were evaluated and 14 (36%) were successfully challenged without any reactions.	Following a negative amoxicillin challenge, antibiotics were modified in 5/14 (36%) patients.
Ward and Fredenrich, 2021^44^	Setting: VAMC in Tampa, FL. Population: Adults admitted with documentation of a penicillin allergy in electronic health records.	Pharmacy team (preceptor and student) interviewed 173 inpatients over 3 yr (2018–2020). Allergies were updated in the electronic medical record for 161 patients. They referred 107 patients to clinic, 23 of whom came to clinic, many for skin testing.	Negative skin test ranged from 63% to 95% of patients. Of those with negative skin tests, only 17%–95% had allergy listing removed (this rate generally increased over the study period).	Patient interviews were performed by fourth-year advanced practice pharmacy students. After negative skin test, phone calls often had to be made to other VA facilities where penicillin allergy was still listed to enable removal in the electronic health record.
Temiño et al., 2018^33^	Setting: VAMC in Miami, FL. Population: Adult outpatients with self-reported penicillin allergy referred to the allergy-immunology clinic for any atopic concern.	Penicillin skin testing quality assurance initiative over 10 mo (2014–2015) with 2-yr follow-up.	In total, 93% of patients had a negative penicillin skin test. In total, 42% of patients with a negative penicillin skin test subsequently took penicillin within the 2-yr follow-up period.	In total, 45% of patients previously prescribed and tolerated cephalosporins. Outpatient testing reduced broad-spectrum antibiotic use by 39% over 3 yr.
Ortiz et al, 2016^31^,^a^	Setting: VAMC in San Antonio, TX. Population: Adults admitted with documentation of new penicillin allergy in the electronic health record.	Pharmacists called patients with self-reported penicillin allergies admitted in the first 3 mo of 2014. Fifty-five patients completed the process.	Portion of patients with nature of reaction documented improved from 54% to 93%. Overall accuracy of documentation improved from 33% to 86%.	Clinical pharmacists called patients after discharge using a standardized questionnaire.
Valente et al., 2007^45^	Setting: VAMC in Los Angeles, CA. Population: Adults (inpatients and outpatients).	Allergy/ADR educational campaign targeting patients and nursing staff over 6 mo (2005). Allergy delisting if and only if direct reconciliation and confirmation with patient and family.	Monthly, nursing ADRs increased from 2 (Feb 2005) to 35 (Sept 2005). In total, 30% of patients reported medication allergies.	In total, 10%–15% of patients had allergies listed in chart that they did not remember.
Klaustermeyer and Gowda, 2005^32^	Setting: VAMC in Los Angeles, CA. Population: Adults (inpatients and outpatients) with a history of immediate-type penicillin allergy and no suitable alternatives following infectious disease consultation.	Penicillin skin testing of 122 patients across 20 yr (1978–1998). Patients with a negative test were eligible for systemic penicillins.	In total, 8% of patients with positive penicillin skin tests.	In total, 4% of patients with negative skin tests had an allergic reaction when given systemic penicillin. None of the reactions were considered life-threatening.

Note. ADR, adverse drug reaction; ASP/ID, antimicrobial stewardship program/infectious diseases; PST, penicillin skin testing; VA, Veterans Affairs; VAMC, VA Medical Center; VHA, Veterans Health Administration.

a Abstract only available.

### Prevalence and impact of unverified penicillin allergy labels within the VHA

The prevalence of allergies to penicillin-class antibiotics among inpatients at non-VHA facilities/systems ranges between 9% and 15%,^2^ and the literature suggests that this is similar within VHA facilities. McConeghy and colleagues evaluated allergy documentation for more than 10 million admissions to any VHA facility between 2000 and 2014, the largest VHA study to date.^15^ They found allergy to one or more members of the penicillin-class of antibiotics to be the most frequently reported (13%), followed by opiates (9.1%), angiotensin-converting enzyme (ACE) inhibitors (5.7%), and sulfonamides (5.1%). For patients who were determined to have an allergy to penicillin-class antibiotics, the most frequently documented reactions were rash (25.5%), hives/urticaria (13.8%), and swelling/edema (5.9%). Anaphylaxis was relatively rare, having been reported in just 3.1% of cases. Notably, documentation of the nature of reactions to penicillin-class antibiotics was missing in 43.3% of cases.

Within the VHA, available data on the impact of the penicillin allergy label are limited to single sites. Conway and colleagues performed a retrospective study at the VA Western New York Healthcare System, which included 403 patients admitted between January 2006 and December 2015 with a diagnosis of pneumonia, urinary tract infection, bacteremia, or sepsis.^16^ The presence of a penicillin allergy label in the medical record was associated with a 50-min delay in the administration of the first dose of antibiotics in the emergency department and significantly higher rates of fluoroquinolone and carbapenem use as initial antibiotic therapy, as compared to those who did not have a penicillin allergy label. Patients with a penicillin allergy label were also significantly less likely than those without such a label to have their antibiotic regimen narrowed. The study authors did not identify any significant differences in total antibiotic duration, length of stay, or 30-d readmission rates between the groups.

Other VA single-site studies have also reported an association between β-lactam allergy (mainly to penicillin-class antibiotics) and suboptimal antibiotic choices both in the outpatient and inpatient settings.^17–19^ However, no consistent adverse signal on infection outcomes was reported in these studies, potentially attributable to the small sample sizes. A 60% average increased antibiotic cost was reported in veterans with penicillin allergy at one VA facility.^20^ In a multisite VA facility study, β-lactam allergy was also the most common reason for vancomycin perioperative prophylaxis in colorectal and vascular procedures.^21^

Importantly, and in contrast to non-VHA health system studies,^22–24^ we found no multisite VHA facility studies examining the impact of penicillin or β-lactam allergy labels on hospital length of stay, mortality, and adverse outcomes for specific infections (e.g., community-acquired pneumonia, surgical site infections, or *Staphylococcus aureus* bacteremia) in the veteran population (Table 1). This highlights an important gap in the literature and one which future studies should seek to address.

### Advancing evaluation of penicillin allergies and delabeling within VHA

#### Access to penicillin allergy evaluations

The VHA Antimicrobial Stewardship Task Force conducted an agency-wide survey on antimicrobial stewardship programs in 2020 that revealed heterogeneity of access to penicillin allergy evaluation services. Among 138 respondent facilities, 28 (20%) reported having a formal process for evaluating patients with a penicillin allergy label, with only 17 of these 28 (61%) reporting ready access to penicillin skin testing (PST).^25^ However, 70 of the total 138 facilities (51%) reported the use of *ad hoc* processes for the evaluation of patients with documented allergies to penicillin-class antibiotics; no further information was reported on the mechanisms of these processes.

Nationally, there is a shortage of board-certified allergists—a well-recognized problem that is only expected to worsen in the coming years.^26^ Although there are no published data that report on staffing levels in the VHA, it is known that many VHA facilities of varying sizes and complexity levels do not have allergy/immunology providers on staff. For those facilities without on-site access to allergy care, access to community (non-VHA) allergy specialists is available through the VA Maintaining Internal Systems and Strengthening Integrated Outside Networks (MISSION) Act. Passed in 2018, the VA MISSION Act aimed to expand the services available to veterans by allowing them access to services in the community, primarily when VHA is unable to provide the service directly because of excessive distance from or an extended wait time at a VHA facility.^27^ Although veterans with penicillin allergy labels enrolled at the majority of VHA facilities would be eligible for this expanded access to care, the successful utilization of this alternative pathway is contingent upon a number of factors, which include the local availability of contracted allergy providers, awareness on the part of the primary care provider to initiate a referral for penicillin allergy evaluation, the ability of the veteran to travel to the contracted allergy provider, reliable propagation of the results of any allergy evaluation back to VHA, and the subsequent modification of the allergy listing in the veteran’s electronic health record. Based on our review of penicillin allergy evaluation within the VHA (Table 2), we were unable to identify a single study examining the impact of the VA MISSION Act or use of community allergy providers for penicillin allergy evaluations.

#### Direct delabeling from chart review

Since the 1970s, the VHA has been a pioneer of electronic health records, with all facilities having implemented the VHA’s Computerized Patient Record System by 1999.^28^ The presence of a standardized and interconnected record allows providers to view the details of patient records, including medication and allergy histories, from any other VHA facility where a patient received care. This record-keeping goes back decades, which is a considerable advantage when evaluating a patient for direct delabeling based on chart review.

At the Memphis VA Medical Center, Mitchell and colleagues implemented a program in which clinical pharmacy specialists evaluated and interviewed patients admitted with β-lactam allergies and then documented their assessments and recommendations: clearance for alternative β-lactams, avoidance of all β-lactams, direct removal of the allergy label, or (beginning January 2019) referral to a pharmacist-led penicillin allergy clinic for PST.^29^ Of 278 patients screened between November 2017 and February 2020, 62 had their allergy labels removed following this program’s evaluation with an additional 180 eligible for alternative beta-lactams following this program’s evaluation.

Gaberino and colleagues at the VA Medical Center in Milwaukee instituted a multidisciplinary (emergency medicine, allergy, internal medicine, and infectious diseases) guideline in November 2017 to guide antibiotic management and β-lactam allergy relabeling for patients with documented β-lactam allergies.^30^ Like Mitchell et al, outcomes included complete allergy label removal, update of the allergy record to support use of other β-lactams, and no change to the label; notably, there was no option for referral to a penicillin allergy clinic. Between November 2017 and February 2020, they identified 79 patients with a β-lactam allergy label (90% penicillins and 10% cephalosporins). Complete removal of the allergy label was feasible for 27% of these patients, resulting in a significant increase in the use of β-lactam antibiotics (44%–77%; *P* < .001).

Evaluation and clarification of penicillin allergies have also occurred asynchronously without the need for inpatient admission or an outpatient clinic. Ortiz and colleagues led an initiative at the South Texas VA Health Care System in San Antonio that utilized a team of pharmacists to improve the accuracy of recently documented penicillin allergies.^31^ They identified 229 patients with new penicillin allergies documented between October 1, 2014, and January 31, 2015, 55 (24%) of which were contacted to complete a short questionnaire regarding their allergy history. This process resulted in the documentation of the nature of the reaction in 93% of cases (compared to 54% in a preintervention cohort), and documentation of other key elements (date and timing of the reaction and history of antibiotic use since the reaction) improved to 66% or better from a preintervention rate of less than 2%. Overall, accuracy improved to 86% postintervention compared with 33% prior to the intervention. Of note, this initiative did not include the removal of penicillin allergy labels, and the lack of long-term follow-up on subsequent antibiotic use limits the understanding of the overall impact of the program.

#### Penicillin skin testing and direct oral amoxicillin challenge

For decades, referral to allergy clinics for PST (with or without subsequent oral amoxicillin challenge) has been the standard approach for evaluating penicillin allergy both outside and within the VHA. In 2005, Klaustermeyer and Gowda reported on the experience of the West Los Angeles VA Medical Center, where PST was performed under a research protocol from September 1978 through May 1998.^32^ Patients were identified from both inpatient and outpatient services, and all of those included had a reported history of reaction to a penicillin-class antibiotic within 48 h of exposure. PST was only performed in consultation with infectious diseases and in cases where no suitable alternative agents were available for treatment. Over the study period, 122 patients underwent PST, of which 110 (90.2%) had their allergy labels removed.

More recently, Temiño and colleagues at the Miami VA Medical Center reported on a successful pilot program to offer routine PST to all patients seen in an outpatient allergy clinic for any reason.^33^ Between May 2014 and March 2015, 40 patients underwent PST and 1 underwent oral amoxicillin challenge without a preceding PST. Of the 40 patients who underwent PST, 38 (93%) had negative reactions and proceeded to oral challenge, only one of whom had a reaction of any sort (fixed drug eruption that developed approximately 10 h following negative PST). Through March 2017, 16 of the 38 patients who had a negative PST received 23 unique courses of penicillin-class antibiotics, a majority of which (57%) were taken by the 10 patients originally referred by the infectious diseases or spinal cord injury services. This program highlights the potential value of targeting efforts to select populations within the VHA who are most likely to need future antibiotic treatments.

In addition to the direct removal of allergy labels following careful evaluation as discussed previously, Mitchell and colleagues also referred select patients to a pharmacist-driven clinic for PST followed by oral amoxicillin challenge.^29^ Of the 32 patients referred, 24 were able to have their allergy labels removed through direct testing. Of the eight patients who could not be delabeled, PST confirmed the allergy in five (62.5%) and was inconclusive in three (37.5%); clearance for alternative β-lactam agents was given and documented in the patients’ charts. At the time of this publication, the authors reported that 147 (80%) of the 184 patients evaluated at the Memphis VA Medical Center who were eligible to be seen in the penicillin allergy clinic were awaiting scheduling for possible PST, highlighting how the use of a clinic-based penicillin evaluation service could create a potential bottleneck.

This model has since been adopted by the VHA’s Innovations Ecosystem (a program to drive forward innovations within the VHA)^34^ and has recently resulted in the launch of a voluntary program termed ABLE—Allergy to Beta-Lactam Evaluation—to assist pharmacists in the evaluation and clarification of penicillin allergies.^35^ The program benefits from multidisciplinary stakeholder collaboration, leveraging the interconnected nature of the VHA computer systems and standardized electronic health records to facilitate rapid implementation of predeveloped templates, protocols, and documentation tools, with minimal required modifications. However, limitations to this strategy include reliance upon a voluntary physician champion (usually one specializing in allergy/immunology) and a dedicated clinical pharmacist as well as the need for clinic space and support staff if performing outpatient evaluations. These are limited resources at many VHA facilities, and securing them to implement and support a new, nonurgent service among a field of other competing services and programs that serve the needs of veterans can be a challenge.

Direct oral amoxicillin challenge (without preceding PST) has emerged as a safe and effective means of delabelling patients with low-risk allergy histories and is supported by a recent randomized controlled trial.^36^ Under this paradigm, patients who are deemed to have low-risk penicillin allergy histories are given a one-time dose of amoxicillin 250–500 mg orally and then observed for 30–60 min; patients who do not have a reaction may then have their penicillin allergy label removed from the chart. A major advantage to this approach is the lower barrier to implementation—in terms of the decreased need for special training in administering PST agents and interpreting the results, the use of a medication product already stocked at most facilities, and a decrease in the time required for each challenge—as compared to PST. Although we are aware of several efforts to implement direct oral amoxicillin challenges within different VHA facilities, including the recently launched ABLE initiative, there are no published, peer-reviewed, VHA system-wide data on the implementation and effectiveness of this approach. Given the endorsement of this approach in the 2022 practice parameter update from AAAAI and ACAAI and the ease of safely implementing this strategy without the need for allergy/immunology specialist oversight, we strongly support this practice and efforts to implement it within the VHA. A sizable proportion of veterans would be eligible for this evaluation, as supported by the literature.

However, penicillin allergy evaluation and direct oral amoxicillin challenge need not be limited to dedicated services, and several efforts have been made to incorporate these activities into pre-existing clinics/services. Using national data from VHA’s Corporate Data Warehouse, Vivo and colleagues performed a retrospective analysis of patients with outpatient dental clinic visits at any VHA between 2015 and 2018.^37^ They identified 26,236 patients who had a dental visit with an associated antibiotic prescription within 7 d of the visit and who had prior documentation of a non-anaphylactic allergy to penicillin; identified prescriptions were predominantly (98%) for non-cephalosporin antibiotics. After analyzing a geographically stratified random sampling of 100 of those patients who did not receive a cephalosporin antibiotic, the authors determined that over a quarter of these patients would have been eligible for direct oral amoxicillin challenge. We have identified limited single-VA facility reports of the use of direct oral amoxicillin challenge as a means of removing penicillin allergies: one report by Nguyen and colleagues at the VA Greater Los Angeles Health Care System focused on patients in the emergency department^38^ and one by Piazza and colleagues at the Miami VA Medical Center detailing their experiences in the inpatient setting.^39^ While the available data indicate that these programs appear to be safe, the overall numbers of patients are small, and more data are needed to truly understand the potential impact and feasibility of such a strategy.

Although the above experiences highlight the advantages of using direct oral amoxicillin challenges to delabel patients with low-risk allergy histories, we have found that implementation of this approach also has some challenges. First, a direct challenge requires patient monitoring for 30–60 min after the challenge dose is administered, which can be hindered by staff availability. Additionally, patient, family member, and healthcare worker beliefs regarding the safety of penicillin allergy removal and adjustment of the medical record present barriers, as reported in a recent VHA study by Gillespie and colleagues.^40^ These challenges highlight why interdisciplinary collaboration, targeted education, and communication are key to ameliorating concerns regarding patient safety and preventing increased workload burdens among frontline staff.

## Discussion

Although much is known about the prevalence of penicillin allergy labels within the VHA, there is a relative paucity of data on the impact of such labels on clinical outcomes for veterans admitted to VHA facilities. Implementation studies within the VHA on either PST or direct amoxicillin challenges have had direct support from internal allergy/immunology providers, which calls into question the scalability of facilities lacking such support. The recent development of a centralized hub of resources to assist pharmacists in penicillin allergy evaluation and removal within the VHA is encouraging, but further prospective studies will be needed to assess its adoption and impact on patient care including cost, length of stay, delays in initiation of appropriate antibiotic treatment, and relabeling. Future studies should also give consideration to contextual barriers, the dependability of the VA MISSION Act to utilize non-VHA-allergy/immunology providers, the use of telemedicine in rural VHA facilities, and the unique challenges presented by vulnerable veteran populations with penicillin allergies, including those who have communication barriers, substance use disorder, or are without housing or reliable transportation.

## Supporting information

Kouma et al. supplementary material Kouma et al. supplementary material

## Supplementary material

For supplementary material accompanying this paper visit http://doi.org/10.1017/ash.2023.448. click here to view supplementary material
