# Supplementary material for: Challenges and opportunities related to penicillin allergy in the Veterans Health Administration: a narrative review
Source: Antimicrob Steward Healthc Epidemiol. 2023 Oct 19;3(1):e174. doi: 10.1017/ash.2023.448 (PMC10644167; doi:10.1017/ash.2023.448)

**Methods**

We reviewed the medical literature to inform this narrative review of penicillin allergy within the US Veteran population. To identify references, we searched the PubMed database using combinations of keywords, including the following Medical Subject Headings: “Drug hypersensitivity,” “Penicillins,” “beta-Lactams,” “United States Department of Veterans Affairs,” “Veterans,” and “Penicillins/adverse effects.” We retrieved English-language articles published between 1970 and 2022 that were readily available through the VA North Texas Library. We retrieved additional references through authors’ subject matter expertise, article citations, and abstracts and presentations from national infectious diseases (IDWeek) and stewardship conferences (SHEA and VHA Antimicrobial Stewardship Taskforce Presentations). Articles were included in the review if they were conducted within the VHA system and were relevant to penicillin and β-lactam allergies (**Supplementary Figure 1**). **Tables 1** and **2** in the main text provide a full list of the articles used in this review.

**Supplemental Figure 1**


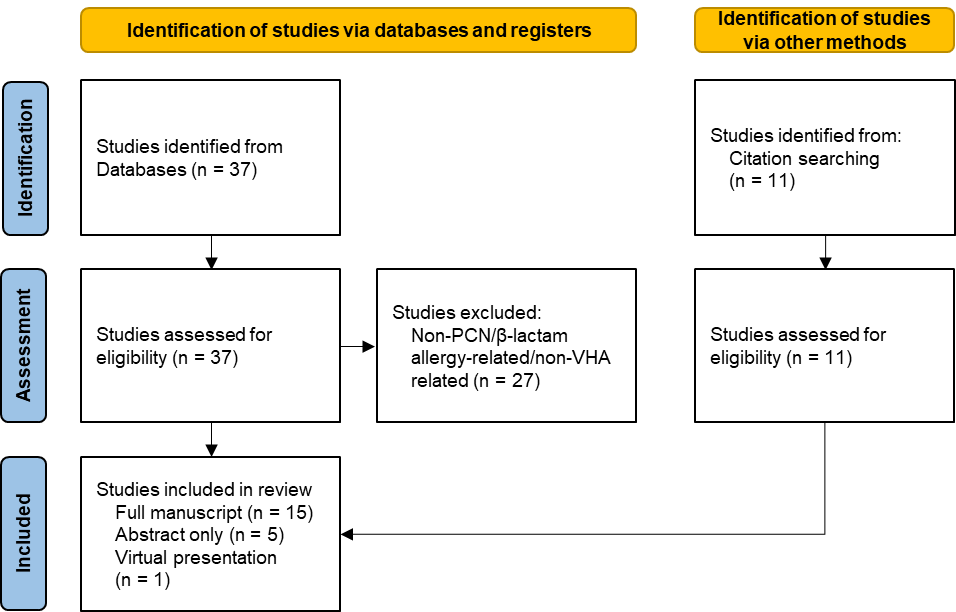

Supplement: Kouma et al. supplementary material [file S2732494X23004485sup001.docx]
